# Medical Debt and Entry of Satellite Freestanding Emergency Departments

**DOI:** 10.1001/jamanetworkopen.2025.22876

**Published:** 2025-07-23

**Authors:** Daniel Marthey, Benjamin Ukert, Elena Andreyeva

**Affiliations:** 1 Department of Health Policy and Management, Texas A&M University, College Station

## Abstract

**Importance:**

Existing evidence suggests the growth of freestanding emergency departments (EDs) is associated with net increases in health care spending and inefficient use of emergency services; however, empirical research examining how these facilities are associated with patients’ financial well-being is lacking.

**Objective:**

To examine whether the entry of hospital-owned satellite freestanding EDs was associated with changes in county-level medical debt in collections.

**Design, Setting, and Participants:**

This cross-sectional study examined the association of the entry of hospital-owned satellite freestanding EDs with medical debt between January 1, 2014, and December 31, 2021, using a difference-in-differences design comparing counties with a freestanding ED opening with counties that did not experience an opening (unexposed). The sample was restricted to counties consistently observed in the Urban Institute Credit Bureau Panel between 2011 and 2021; counties with an existing freestanding ED on or before 2014 were excluded from analysis. Data analysis was conducted between August 2024 and April 2025.

**Exposure:**

Entry of freestanding EDs between 2015 and 2021.

**Main Outcomes and Measures:**

The median medical debt in collections (among individuals with medical debt in collections) was the main outcome measure. Freestanding ED entry and medical debt outcomes were measured on the county-year level. County-by-year data on medical debt came from the Urban Institute Credit Bureau Panel, and data on freestanding ED openings came from the Centers for Medicare & Medicaid Services Provider of Services File.

**Results:**

The study sample included 1368 counties (48 exposed, 1320 unexposed) observed between 2011 and 2021. The baseline characteristics of exposed and unexposed counties suggested that counties exposed to freestanding EDs, on average, had a larger proportion of younger residents (≤17 years, 23.9% vs 22.8%; *P* = .01; ≥65 years, 15.0% vs 16.5%; *P* = .007), had a higher median household income ($53 284 vs $46 535; *P* < .001), and were more likely to be designated as urban (75.0% vs 35.1%; *P* < .001). Freestanding ED entry was associated with an increase of $98.20 (95% CI, $17.60-$178.81) in county-level median medical debt in collections and a 2.0–percentage point increase (95% CI, 0.7-3.2 percentage points) in the proportion of the population with medical debt in collections.

**Conclusions and Relevance:**

In this cross-sectional study, freestanding ED entry was associated with increased medical debt. Additional research is needed to better understand the mechanisms to aid the development of policies aimed at protecting patients’ financial health.

## Key Points

**Question:**

Is the entry of satellite freestanding emergency departments (EDs) associated with changes in medical debt in collections?

**Findings:**

In this cross-sectional study with difference-in-differences analysis, the entry of satellite freestanding EDs was associated with a statistically significant increase of $98 in median medical debt in collections and a 2–percentage point increase in the share of the population with medical debt.

**Meaning:**

This study suggests that policymakers should weigh the benefits associated with improved access to outpatient emergency services with the potential long-term implications for patient financial well-being.

## Teaser

This cross-sectional study examines whether the entry of hospital-owned satellite freestanding emergency departments was associated with changes in county-level medical debt in collections.

## Introduction

Medical debt represents more than 60% of all debt in collections, and nearly 20 million US adults have medical debt.^1^ Individuals with low income and those who report health problems are more likely to have medical debt.^2^ Although health insurance coverage offers some financial protection against incurring medical expenses and thereby medical debt, insurance plan type, design, and provider networks vary considerably, leaving some patients, including those with private insurance, at increased risk of out-of-pocket medical spending, particularly during health emergencies.^3,4,5^ Furthermore, a growing number of outpatient medical care institutions charge facility fees or have relied on balance billing, thereby increasing the cost for services, which may lead to unanticipated (and out-of-network) bills and, potentially, medical debt.^6,7,8,9^

One form of medical institution where facility charges are a common feature are satellite freestanding emergency departments (EDs), which have been known to use suspect advertising practices, passing themselves off as alternatives to urgent care clinics (even though they charge prices similar to hospital EDs) and putting patients at risk of noncovered medical bills.^10^ These satellite facilities are owned and operated by a hospital that provides emergency services at a location that is distinct from the main hospital campus. Freestanding EDs must follow federal requirements for provider-based status outlined in 42 CFR 413.65. For example, they must be located within 56 km (35 miles) of the main hospital campus, and the services provided at freestanding EDs must be fully integrated with the main hospital, including staff with privileges at the main hospital campus. If the affiliated hospital is certified by the Centers for Medicare & Medicaid Services (CMS), the facility is subject to Emergency Medical Treatment and Labor Act standards and must also meet other conditions for participation in Medicare and Medicaid.

The proliferation of freestanding EDs and their billing practices have garnered state and national attention due to high and often noncovered medical bills.^11,12,13,14,15^ In states such as Texas, where freestanding ED facilities represent one-fourth of all ED use, legislators responded by increasing regulation to reduce deceptive practices and to increase price transparency for consumers.^11^ For example, the Texas House Bill 2041 (2019) prohibited certain advertising practices and required the posting of notices to inform patients about their status as a freestanding ED, their median observation and facility fees, and any health benefit plans in which they are a network medical institution. This was followed by the federal No Surprises Act in 2022, which prohibited the practice of balance billing by facilities and medical institutions for emergency medical services. However, these laws may not go far enough to prevent unexpected medical bills.^15^

The growth of freestanding EDs could influence the share of people with medical debt and the amount of medical debt due to the high cost of care at freestanding ED facilities. Recent empirical work has shown that the entry of freestanding EDs increased the use of emergency care without reducing congestion at nearby hospital-based EDs.^13^ In addition, if freestanding EDs act as substitutes for services in urgent care settings, insured patients would be subjected to out-of-pocket costs on a bill 10 times larger than what would be expected for the same treatment in an urgent care facility.^16^ To our knowledge, there is no current empirical evidence of the association between freestanding EDs and medical debt; this study aimed to fill this gap.

## Methods

This study was deemed to be exempt from review by the institutional review board at Texas A&M University because it did not involve human subjects research. This study followed the Strengthening the Reporting of Observational Studies in Epidemiology (STROBE) reporting guideline.

### Data and Study Population

This study used county-level location information on freestanding EDs obtained from the CMS Provider of Services (POS) files from 2011 to 2021. The data are generated by the CMS certification process and contain information on all CMS participating hospitals (and other medical institutions), including hospital facility names and locations, participation and termination dates, facility type indicators, and services. In 2014, the CMS began reporting the number of off-site ED locations (ie, freestanding EDs) operated by hospital facilities. Exact locations of freestanding EDs are not reported; however, federal regulations require off-site ED facilities to be located within 56 km (35 miles) of the main campus, and previous research has found that most of these facilities are located within 9.6 km (6 miles) of the nearest hospital facility.^17^ For our analysis, we restricted the POS file to short-term acute care hospitals and calculated the number of reported freestanding ED facilities for each county based on the location of the main hospital campus reporting off-site freestanding EDs. We cross-referenced the location of freestanding ED openings in the POS file using searches for news articles, press releases, and facility licensure data from the Texas Department of State Health Services.

Data on county-level medical debt outcomes come from the publicly available Urban Institute Credit Bureau Panel.^18^ This dataset provides information on county-level medical debt and is derived from a 2% random sample of US adult consumers for each year, representing approximately 5 million consumers per year. We restricted the file to counties consistently represented in the data between 2011 and 2021. We further excluded counties that had existing satellite freestanding EDs on or before 2014 to avoid bias arising from comparing always-treated counties with later-treated counties (eMethods 1 and eFigure 1 in Supplement 1).^19^

### Outcomes

Outcomes were measured at the county-year level and include the median amount of medical debt in collections (among individuals with medical debt in collections) and the share of adults with a credit history who have medical debt in collections.

### Statistical Analysis

Statistical analyses were conducted between August 2024 and April 2025. We used difference-in-differences models that compared changes in medical debt between counties operating at least 1 freestanding ED during the study time frame and counties in which a freestanding ED facility never opened. Coefficient estimates can be interpreted as the mean difference in medical debt outcomes between exposed and unexposed counties after freestanding ED entry compared with the period before entry. In this study, counties experienced freestanding ED openings at different times (2015-2021). Primary specifications used a doubly robust inverse probability weighted difference-in-differences approach implemented using the Callaway & Sant’Anna^20^ estimator. The approach estimates, in a first stage, the propensity score using county characteristics and then separately estimates the intent-to-treat effect of the association between freestanding EDs and medical debt for each county-group cohort (defined by year of exposure), overcoming the limitations of 2-way fixed effects in settings with variation in treatment timing.^20,21^

The probability weight in the first stage of a freestanding ED opening was estimated using county urban status, the proportion of the population who were uninsured, the median household income, the hospital market concentration, the total population size, and an indicator for Medicaid expansion status. The outcome regression added the county unemployment rate and the estimated distribution of the population by age (18-64 years and ≥65 years) and race and ethnicity (Hispanic, non-Hispanic Black, and non-Hispanic White), derived from self-reported bridged race and ethnicity estimates in the US Census. We included the racial and ethnic composition of counties to adjust for differential experiences with medical debt. We report robust SEs clustered at the county level.^22^

Covariate data were collected from the US Census Bureau’s Small Area Health Insurance Estimates^23^ and Small Area Income and Poverty Estimates^24^ programs; the National Cancer Institute Surveillance, Epidemiology, and End Results Program^25^; and the US Bureau of Labor Statistics Local Area Unemployment Statistics.^26^

The identifying assumption of the difference-in-differences approach is that in the absence of freestanding ED entry, treatment and control counties would have followed similar trends in medical debt outcomes, controlling for time-invariant and time-variant differences among counties nested within states. We evaluated the assumption by estimating event-study models to examine the evolution of treatment effects on medical debt around the year of freestanding ED entry into counties (Figure).^20^ As sensitivity tests, we also provide estimates from a stacked difference-in-differences approach^27^ and examine the association between freestanding ED entry and medical debt across the intensity of treatment, measured as the total number of freestanding ED openings.

**Figure.**
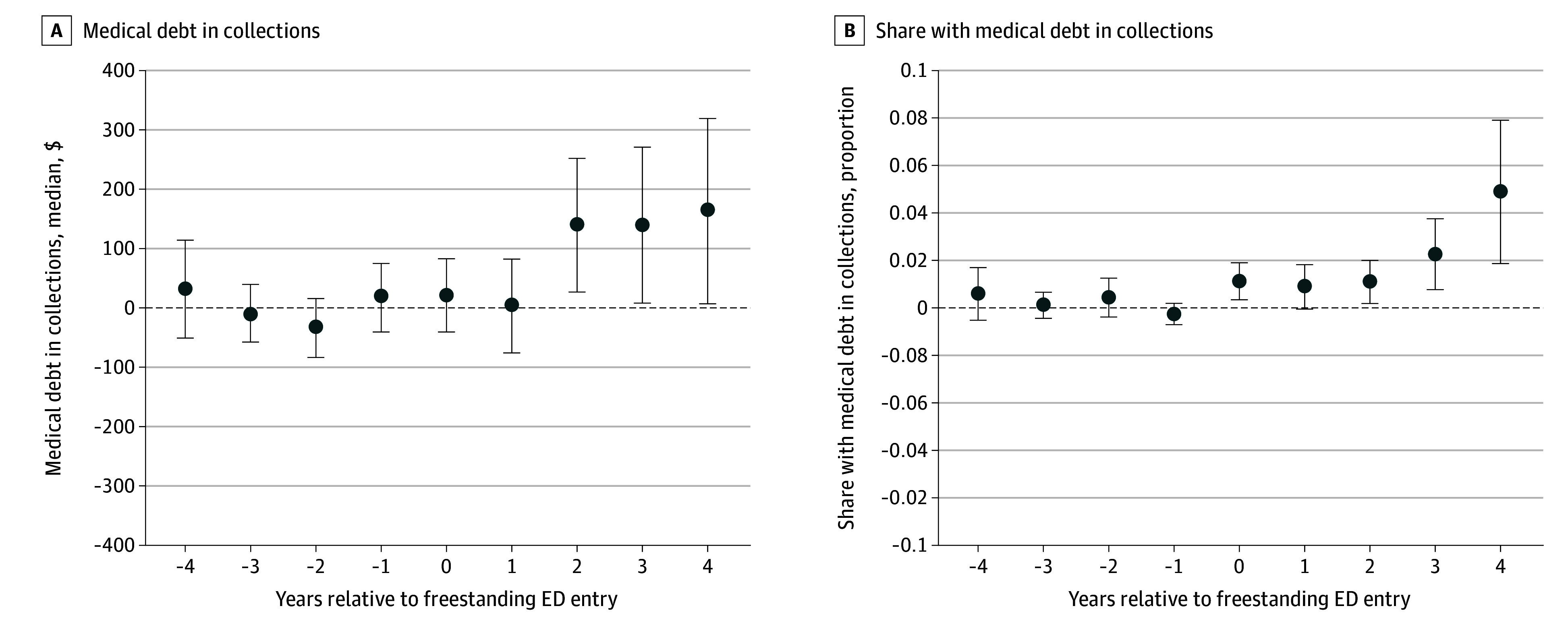
Event-Study Estimates From a Doubly Robust Inverse Probability Weighted Approach Implemented Using the Callaway & Sant'Anna^20^ Estimator The propensity scores were estimated using county urban status, the percentage uninsured, median household income, hospital market concentration, the total population count, and Medicaid expansion status. The outcome regression added the unemployment rate, the distribution of the population by age (% aged 18-64 years and % aged ≥65 years), and race and ethnicity (% Hispanic, % non-Hispanic Black, and % non-Hispanic White). Error bars indicate 95% CIs. ED indicates emergency department.

More details regarding study design, statistical analyses, and robustness tests for the stacked estimator can be found in the eMethods 2 and eTable 2 in Supplement 1. Statistical analyses were conducted using Stata/MP software, version 18.5 (StataCorp LLC). Two-sided tests were considered significant at *P* ≤ .05.

## Results

### Descriptive Statistics

The study included 1368 counties, with 48 counties exposed to freestanding EDs between 2015 and 2021. The sample includes counties from all states except Delaware, Minnesota, Nebraska, Rhode Island, and South Dakota. A graphical representation of sample definitions is provided in eFigure 1 in Supplement 1. Unweighted baseline characteristics of counties are displayed in Table 1, and weighted summary statistics are displayed in eTable 1 in Supplement 1. The distribution of the population by age was similar in counties by future freestanding ED exposure status, although some estimates were statistically different from zero. For example, counties that experienced a freestanding ED opening had a larger proportion of Hispanic residents (freestanding ED exposure, 14.5% vs no freestanding ED exposure, 9.3%; *P* = .01) (Table 1). Both groups had similar proportions of non-Hispanic Black residents (freestanding ED exposure, 9.7% vs no freestanding ED exposure, 11.7%; *P* = .36). Exposed counties, on average, had a higher median household income (freestanding ED exposure, $53 284 vs no freestanding ED exposure, $46 535; *P* < .001), were significantly more likely to be designated as urban (freestanding ED exposure, 75.0% vs no freestanding ED exposure, 35.1%; *P* < .001), and had a larger proportion of younger residents (≤17 years: freestanding ED exposure, 23.9% vs no freestanding ED exposure, 22.8%; ≥65 years: freestanding ED exposure, 15.0% vs no freestanding ED exposure, 16.5%). The 2 groups were similar in terms of the mean hospital market concentration measured as the Herfindahl-Hirschman Index (freestanding ED exposure, 2420 vs no freestanding ED exposure, 2461; *P* = .84) and the baseline median medical debt in collections (freestanding ED exposure, $932 vs no freestanding ED exposure, $951; *P* = .70), but exposed counties had a higher baseline share of the population with medical debt in collections (freestanding ED exposure, 22.0% vs no freestanding ED exposure, 26.0%; *P* = .001).

**Table 1.** Unweighted Baseline Characteristics of Counties by Exposure to Freestanding EDs, 2014^a^

Characteristic	No freestanding ED exposure	Freestanding ED exposure	Standardized difference	*P* value
No. of counties	1320	48	NA	NA
Age, %				
≤17 y	22.8	23.9	−0.350	.01
18-64 y	60.7	61.2	−0.143	.34
≥65 y	16.5	15.0	0.406	.007
Race and ethnicity, %				
Hispanic	9.3	14.5	−0.311	.01
Non-Hispanic Black	11.7	9.7	0.158	.36
Non-Hispanic White	76.1	70.7	0.269	.06
Median household income, $	46 535	53 284	−0.457	<.001
Population at or below 100% FPL, %	17.7	15.4	0.376	.01
Unemployment, %	6.7	5.9	0.466	.004
Uninsured, %	14.7	14.6	0.014	.91
Urban designation, %	35.1	75.0	−0.872	<.001
Mean (SD) Herfindahl-Hirschman Index	2461 (1421)	2420 (1304)	0.030	.84
Median medical debt in collections, $	951	932	0.061	.70
Share with medical debt in collections, %	26.0	22.0	0.498	.001

Abbreviations: ED, emergency department; FPL, federal poverty level; NA, not applicable.

^a^ Freestanding ED locations and county urban or rural designation were obtained from the Centers for Medicare & Medicaid Services Provider of Services files. Hospital Herfindahl-Hirschman Index and medical debt outcomes were obtained from the Urban Institute Credit Bureau Panel (2011-2021). County by year estimates of the population by age and race and ethnicity were obtained from the National Cancer Institute Surveillance, Epidemiology, and End Results Program. Estimates of health insurance coverage, household income, and poverty come from the US Census Bureau. County unemployment was obtained from the US Bureau of Labor Statistics. The sample includes a balanced panel of counties consistently observed between 2011 and 2021. Counties inconsistently observed from the Credit Bureau Panel and counties exposed to satellite freestanding EDs on or before 2014 were excluded from the sample. Freestanding ED exposure indicates a freestanding ED opening in the county between 2015 and 2021, reported to CMS by general acute hospital facilities.

### Difference-in-Differences and Event-Study Estimates

Table 2^20^ shows difference-in-differences estimates of the association between satellite freestanding EDs and medical debt outcomes for our primary specification and an alternative stacked difference-in-differences approach that does not incorporate propensity score weights. Our primary results suggest that satellite freestanding EDs were associated with an increase of $98.20 (95% CI, $17.60-$178.81) in median medical debt in collections and a 2.0–percentage point (pp) increase (95% CI, 0.7-3.2 pp) in the share of the population with a credit history with medical debt in collections. Estimates of changes in medical debt are in broad agreement across the 2 specifications; however, we did not observe changes in the share with medical debt in collections in the alternative stacked regression.

**Table 2.** Difference-in-Differences Estimates of the Association Between Satellite Freestanding Emergency Department Entry and County-Level Medical Debt^a^

Approach	Coefficient (SE) [95% CI]		No.
	Median medical debt, $	Share with medical debt	
Primary specification	98.20 (41.13) [17.60 to 178.81]^b^	0.020 (0.006) [0.007 to 0.032]^b^	15 048
Stacked difference-in-differences	66.42 (33.48) [0.78 to 132.06]^c^	0.005 (0.004) [−0.003 to 0.012]	102 168

^a^ The primary specification is a doubly robust inverse probability weighted approach implemented using the Callaway & Sant’Anna^20^ estimator. The stacked difference-in-differences specification included no weights and clustered SEs on the county by stack. The propensity scores in the primary specification were estimated using county urban status, the percentage uninsured, median household income, hospital market concentration, the total population count, and Medicaid expansion status. Source: Centers for Medicare & Medicaid Provider of Services files and Urban Institute Credit Bureau Panel (2011-2021).

^b^ *P* < .01.

^c^ *P* < .05.

Event-study results in the Figure, A and B,^20^ show no evidence of differential pretrends—baseline estimates are small, and the 95% CIs do not exclude zero. There was a 15% increase in the median medical debt in collections beginning 2 years after entry ($139.45; 95% CI, $27.98-$250.91). Estimates were as large as $163.62 (95% CI, $7.86-$319.38), a 17% increase in median medical debt collections by year 4 after entry. Changes in the share of the population with medical debt appeared in the year of freestanding ED entry, with an increase of 1.1 pp (95% CI, 0.3-1.8 pp). By the fourth year after freestanding ED entry, the share of the population with medical debt increased by 4.8 pp (95% CI, 1.8-7.9 pp).

We examined the association between freestanding ED entry and changes in medical debt in the context of the magnitude of county-level freestanding ED exposure (Table 3).^20^ We defined the magnitude of exposure based on the total number of freestanding EDs that opened in the county during the period of study (ending in 2021). Among counties in the 75th percentile of total satellite freestanding EDs (5 freestanding ED facilities), the difference-in-differences estimate suggested that the opening of a freestanding ED was associated with a $100.72 increase (95% CI, −$135.15 to $336.58) in median medical debt and a 0.8-pp increase (95% CI, −1.7 to 3.3 pp) in the share of the population with medical debt. However, neither estimate reached statistical significance. Among counties in the 95th percentile or above (≥9 freestanding ED facilities), freestanding ED entry was associated with a $577.66 increase (95% CI, $236.69-$918.64) in median medical debt and a 3.2-pp increase (95% CI, –2.0 to 8.4 pp) in the share of the population with medical debt, although the latter did not reach statistical significance.

**Table 3.** Difference-in-Differences Estimates of the Association Between Satellite Freestanding ED Entry and County-Level Medical Debt, by Treatment Intensity^a^

Sample	Coefficient (SE) [95% CI]		No.
	Median medical debt, $	Share with medical debt	
Main result	98.20 (41.13) [17.60 to 178.81]^b^	0.020 (0.006) [0.007 to 0.032]^b^	15 048
75th Percentile total freestanding EDs	100.72 (120.34) [−135.15 to 336.58]	0.008 (0.013) [−0.017 to 0.033]	14 311
95th percentile total freestanding EDs	577.66 (173.97) [236.69 to 918.64]^b^	0.032 (0.027) [−0.020 to 0.084]	14 135

Abbreviation: ED, emergency department.

^a^ The primary specification is a doubly robust inverse probability–weighted approach implemented using the Callaway & Sant’Anna^20^ estimator. Among counties with at least 1 satellite freestanding ED the 75th percentile of total freestanding EDs is 5 facilities. The 95th percentile is 9 or more freestanding ED facilities. Source: Centers for Medicare & Medicaid Provider of Services files and Urban Institute Credit Bureau Panel (2011-2021).

^b^ *P* < .01.

## Discussion

We examined the association between freestanding ED entry and medical debt using data from the CMS POS file and the Urban Institute Credit Bureau Panel. Our intent-to-treat estimates were robust to alternative specifications and suggested that freestanding EDs are associated with a $98 increase in median medical debt in collections and a 2-pp increase in the share of the population with medical debt in collections relative to counties never exposed to freestanding EDs.

Our event studies showed that the increase in medical debt was delayed to 2 years after opening, while changes in the share of the population with medical debt occured immediately. Comparing the magnitude relative to the median medical debt in collections revealed an increase in median medical debt in collections of approximately 15% two years after freestanding ED opening and 17% after 4 years. The share of people with medical debt in collections also remained flat until 3 years after the freestanding ED opening and increased substantially in year 4. The delay in outcomes is consistent with the expected time lag of medical debt reporting, where use is followed by nonpayment, delinquency, and invoice sale to collections.^28^

Studies have suggested that high prices, due to hospital consolidation, may increase patient financial risk.^29,30^ Although individuals with less generous health insurance benefits are at greater risk, those with health insurance coverage are not immune to health-related financial shocks.^3,5^ To account for differences in county composition, we addressed concerns about differential trajectories of medical debt by using a propensity score–weighted approach to adjust for differences in hospital market concentration and other factors associated with county economic shocks and controlled for additional differences in socioeconomic position and population demographics in the outcome model of our primary specifications. The attenuated findings on the association with medical debt and the share of the population with medical debt in collections for the stacked difference-in-difference approach may stem from the fact that we did not use inverse probability weights. As such, we weighted each cohort intent-to-treat effect equally across counties. Furthermore, comparison counties were also equally weighted, which may have increased the influence of outlier counties that were less representative of the median county. Alternatively, the findings may also imply that the increase in medical debt was concentrated among counties with existing high shares of medical debt, exacerbating financial trouble for an already marginalized and/or underinsured population.

The mechanisms of the increase in medical debt are important but not well understood. The association that we observed could be due to individuals who otherwise may consider seeking care at urgent care clinics, who decide to seek care at freestanding EDs after regular business hours when primary care offices are closed. This hypothesis is supported by evidence from Hamer et al,^31^ who used a time-series approach to study visit volumes to an urgent care center after the opening of a freestanding ED in North Baton Rouge, Louisiana. Their results suggested that after the freestanding ED opened, emergency visits to the urgent care center decreased, while there was no change in nonemergency visits. The proportion of nonemergency visits at the freestanding ED increased by approximately 8 pp (*P* < .001) after hours when the urgent care center was closed. Seeking care at freestanding EDs may then lead to large and unexpected medical bills because patients are unaware that these freestanding EDs charge similar rates as hospital EDs. Last, many freestanding EDs are owned by hospital systems, thereby serving as an entry point for subsequent system-owned referrals for outpatient and inpatient services that may result in additional unanticipated out-of-pocket costs.

### Limitations

Our study has several limitations. First, we identified satellite freestanding EDs using the CMS POS file, restricting our analysis to CMS-certified medical institutions; thus, our satellite freestanding ED counts likely represent an undercount of total number of facilities, as a small number of hospital facilities do not participate in Medicare. To our knowledge, no national registry of satellite freestanding EDs exists. Second, we did not observe the physical address of satellite freestanding ED locations. We relied on a federal statute that requires these facilities to be located within 56 km (35 miles) of the affiliated hospital and assigned freestanding EDs to the county in which the reporting hospital was located. Previous evidence has suggested that most freestanding EDs are located with 9.6 km (6 miles) of the nearest hospital facility.^17^ To minimize misclassification, we then searched for news articles, press releases, and facility information to verify county location. Third, Colorado, Delaware, Rhode Island, and Texas have allowed the licensure of independent freestanding EDs.^32^ These facilities are similar to satellite freestanding EDs but, unlike their hospital-affiliated counterparts, are not recognized as EDs by the CMS and are therefore unable to bill Medicare and Medicaid. Delaware and Rhode Island were excluded during sample construction. To overcome bias from the potential association of these facilities with medical debt outcomes, we provide estimates from an analysis further excluding Colorado and Texas (eTable 2 in Supplement 1). We show that the exclusion of these states has very little association with our results. Fourth, in this study we did not observe health care use that could account for changes in medical debt. Other factors, such as differential enrollment in high-deductible health plans, that vary consistent with freestanding ED entry may confound our analysis.

## Conclusions

To our knowledge, this pooled cross-sectional study is the first to examine the association between satellite freestanding ED entry and medical debt. Our results underscore the need for policy and practice to weigh the potential benefits associated with improved access to outpatient emergency care with the potential long-term implications of burdening people with higher medical debt.

## Notes

Supplement 1. **eMethods 1.** Sample Selection **eFigure 1.** Sample Definition by County **eMethods 2.** Robustness **eTable 1.** Baseline Characteristics of Counties by Exposure to Freestanding Emergency Departments (2014), Before and After Applying Inverse Probability Weights **eFigure 2.** Event Study Estimates of the Association Between Satellite Freestanding ED Entry and Median Medical Debt in Collections, Stacked Difference-in-Differences Approach **eTable 2.** Difference-in-Differences (DiD) Estimates of the Association Between Satellite Freestanding Emergency Departments Entry and County-Level Medical Debt, Robustness to Excluding States That Allow Independent Freestanding ED Licensure
eReferences.

Supplement 2.
Data Sharing Statement
