# Supplementary material for: Medical Debt and Entry of Satellite Freestanding Emergency Departments
Source: JAMA Netw Open. 2025 Jul 23;8(7):e2522876. doi: 10.1001/jamanetworkopen.2025.22876 (PMC12287832; doi:10.1001/jamanetworkopen.2025.22876)
Supplement: Supplement 1. — eMethods 1. Sample Selection eFigure 1. Sample Definition by County eMethods 2. Robustness eTable 1. Baseline Characteristics of Counties by Exposure to Freestanding Emergency Departments (2014), Before and After Applying Inverse Probability Weights eFigure 2. Event Study Estimates of the Association Between Satellite Freestanding ED Entry and Median Medical Debt in Collections, Stacked Difference-in-Differences Approach eTable 2. Difference-in-Differences (DiD) Estimates of the Association Between Satellite Freestanding Emergency Departments Entry and County-Level Medical Debt, Robustness to Excluding States That Allow Independent Freestanding ED Licensure eReferences. [file jamanetwopen-e2522876-s001.pdf]

Supplementary Online Content

Marthey D, Ukert B, Andreyeva E. Medical debt and entry of satellite freestanding emergency departments. *JAMA Netw Open.* 2025;8(7):e2522876. doi:10.1001/jamanetworkopen.2025.22876

eMethods 1. Sample Selection

eFigure 1. Sample Definition by County

eMethods 2. Robustness

eTable 1. Baseline Characteristics of Counties by Exposure to Freestanding Emergency Departments (2014), Before and After Applying Inverse Probability Weights

eFigure 2. Event Study Estimates of the Association Between Satellite Freestanding ED Entry and Median Medical Debt in Collections, Stacked Difference-in-Differences Approach

eTable 2. Difference-in-Differences (DiD) Estimates of the Association Between Satellite Freestanding Emergency Departments Entry and County-Level Medical Debt, Robustness to Excluding States That Allow Independent Freestanding ED Licensure

eReferences.

This supplementary material has been provided by the authors to give readers additional information about their work.

© 2025 Marthey D et al. *JAMA Network Open.*

eMethods 1. Sample Selection

To construct our analysis sample, we first restricted the Credit Bureau Panel data to all counties that were consistently reported between 2011 and 2021 (n=1,794). Estimates from counties with 50 or fewer observations are not released—excluding sparsely populated counties from analysis. We then combined the county-by-year data on medical debt with county-by-year indicators of satellite freestanding EDs from the CMS Provider of Services files.

To obtain annualized county-level counts of freestanding EDs we restricted each year (2014-2021) of the POS file to general short term acute hospitals using provider category and subtype indicators. Next, to avoid counting facilities owned by hospitals no longer in operation we excluded any facility that was not eligible to participate in Medicare and those with a non-missing termination or expiration date as of the current year of the file. Facilities from U.S. territories were excluded. Based on CMS requirements, hospitals that seek to operate off-campus emergency departments must demonstrate compliance with hospital Conditions of Participation (CoPs) and provider-based regulations (42 CFR 413.65) through the survey and certification process with CMS[1]—the process through which the POS data are generated. We cross referenced the location of freestanding ED openings in the POS using searches for news articles, press releases, and facility licensure data from the Texas Department of State Health Services.

Our identification strategy relies on observing at least one baseline period; thus, we restricted the sample to exclude counties with existing satellite freestanding EDs on or before 2014 (n=426). Our final analytic sample includes 48 counties that experienced freestanding ED openings between 2015 and 2021 and 1,320 counties that did not experience a freestanding ED opening over the period. We display county-level treatment assignments in eFigure 1, below. Our sample includes counties from all states except Delaware, Minnesota, Nebraska, Rhode Island, and South Dakota.

© 2025 Marthey D et al. *JAMA Network Open.*

eFigure 1. Sample Definition by County

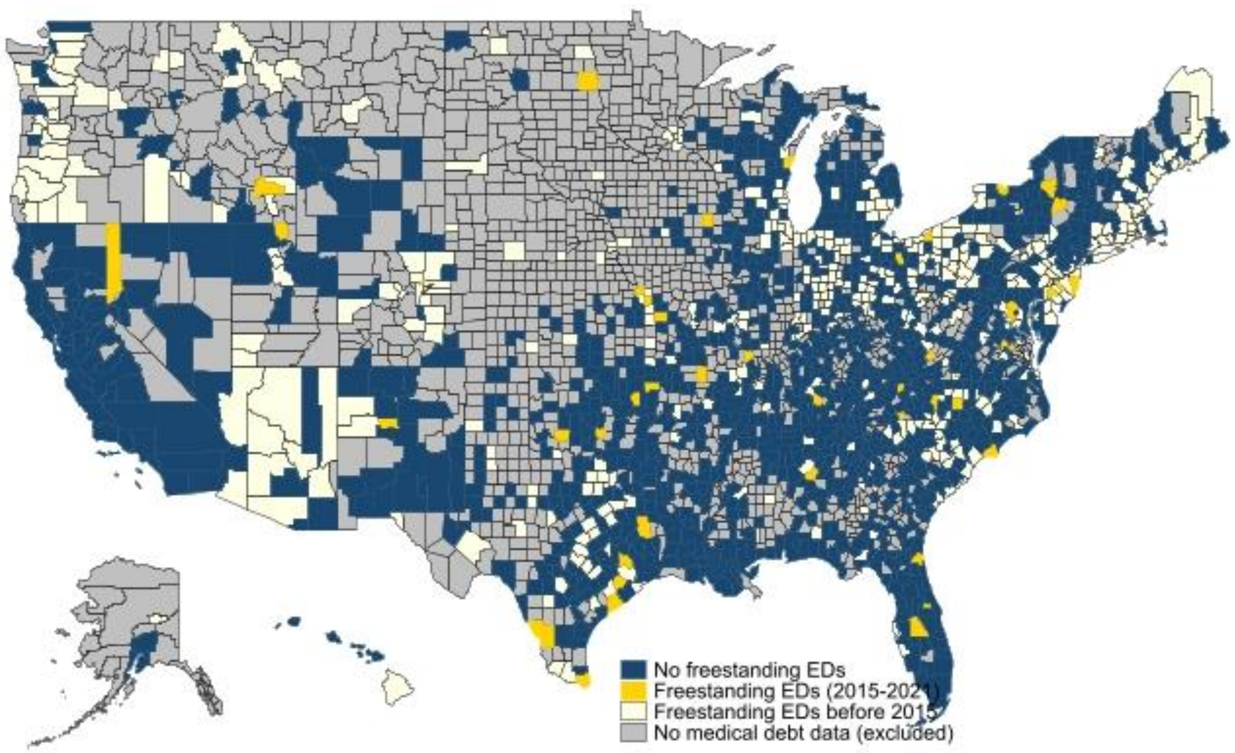

Source(s): Centers for Medicare & Medicaid (CMS) Provider of Services (POS) Files and Urban Institute Credit Bureau Panel (2011-2021).

eMethods 2. Robustness

2.1 Weighted baseline characteristics

In eTable 1 we provide average baseline characteristics before and after applying inverse probability weights where the weight equals 1 for counties exposed to freestanding EDs and counties never exposed to freestanding EDs received a weight equal to the propensity score divided by 1 minus the propensity score. The propensity score model used county urban status, the percentage uninsured, median household income, hospital market concentration, the total population count, and an indicator for Medicaid expansion averaged over the base period to predict freestanding ED entry. After adjustment we observe no meaningful differences in the baseline characteristics of exposed and unexposed counties suggesting observable factors that influence hospital consolidation (and medical debt) should not be a major source of confounding in our analysis.

© 2025 Marthey D et al. *JAMA Network Open.*

eTable 1. Baseline Characteristics of Counties by Exposure to Freestanding Emergency Departments (2014), Before and After Applying Inverse Probability Weights

	**No Adjustment**			**IPW Adjustment**		
Characteristic	No Freestanding ED Exposure	Freestanding ED Exposure	P-Value	No Freestanding ED Exposure	Freestanding ED Exposure	P-Value
Age (%)						
0-17 Years	22.78	23.88	0.011	23.14	23.88	0.13
18-64 Years	60.74	61.18	0.34	61.47	61.18	0.52
65 + Years	16.50	14.95	0.007	15.40	14.95	0.42
Race/Ethnicity (%)						
NH-Black	11.67	9.68	0.36	12.07	9.68	0.11
NH-White	76.05	70.65	0.06	71.26	70.65	0.84
Hispanic	9.30	14.45	0.010	12.32	14.45	0.45
Median Household Income ($)	46,535	53,284	<0.001	53,595	53,284	0.90
Population at or below 100% FPL (%)	17.66	15.42	0.010	15.68	15.42	0.77
Unemployment (%)	6.69	5.88	0.004	6.35	5.88	0.040
Uninsured (%)	14.68	14.60	0.91	14.30	14.60	0.72
Urban Designation (%)	35.08	75.00	<0.001	75.00	75.00	1.00
Mean Herfindahl-Hirschman Index (HHI)	2,461	2,420	0.84	2,398	2,420	0.91
Median Medical Debt in Collections ($)	951	932	0.70	917	932	0.73
Share with Medical Debt in Collections	26.02	21.98	0.001	23.61	21.98	0.16

Notes: Freestanding emergency department (ED) locations and county urban/rural designation were obtained from the Centers for Medicare & Medicaid Services (CMS) Provider of Services (POS) Files. Hospital Herfindahl-Hirschman Index (HHI) and medical debt outcomes from the Urban Institute Credit Bureau Panel (2011-2021). County by year estimates of the population by age and race/ethnicity were obtained from the National Cancer Institute Surveillance, Epidemiology, and End Results (SEER) Program. Estimates of health insurance coverage, household income, and poverty come from the U.S. Census Bureau. County unemployment was obtained from the U.S. Bureau of Labor Statistics. The sample includes a balanced panel of counties consistently observed between 2011 and 2021. Counties inconsistently observed from the Credit Bureau Panel and counties exposed to satellite freestanding EDs on or before 2014 were excluded from the sample. Freestanding ED exposure indicates a facility opening in the county between 2015 and 2021, reported to CMS by general acute hospital facilities. The propensity score model used to calculate the inverse-probability weight (IPW) included county urban status, percent uninsured, median household income, hospital market concentration, the total population count, and an indicator for Medicaid expansion status. NH=non-Hispanic.

© 2025 Marthey D et al. *JAMA Network Open.*

2.2 Alternative difference-in-differences estimator:

Goodman-Bacon (2021) and others have shown that in a staggered difference-in-differences design, the average post-period estimate is a variance weighted average of individual two-by-two difference-in-differences estimators, leading to the possibility of negative weights if treatment effects vary across adoption groups (identified by the period of exposure to the policy) when already treated groups are compared against later adopters.[2] Our primary specification, Callaway & Sant'Anna (2021), avoids these forbidden comparisons by separately estimating group average treatment effects on the treated (ATTs) based on the year of adoption and using never treated units as controls.[3] In robustness we show estimates coming from a stacked difference-in-difference approach that similarly estimates group time ATTs but does so by balancing each exposure period with an unexposed control group through the creation of "stacks".[4]

Average post-period estimates from the stacked difference-in-difference approach are provided in the main manuscript (Table 2). In eFigure 2, below, we display event study estimates for median medical debt in collections. We observe no evidence of differential pre-trends at baseline. Estimates are small and none exclude zero. We observe increases in median medical debt, as large as $100 after three years, but none of the post-period estimates are statistically significant. The magnitude of the period-specific estimates is consistent with those presented in Figure 1 of the main manuscript.

© 2025 Marthey D et al. *JAMA Network Open.*

eFigure 2. Event Study Estimates of the Association Between Satellite Freestanding ED Entry and Median Medical Debt in Collections, Stacked Difference-in-Differences Approach

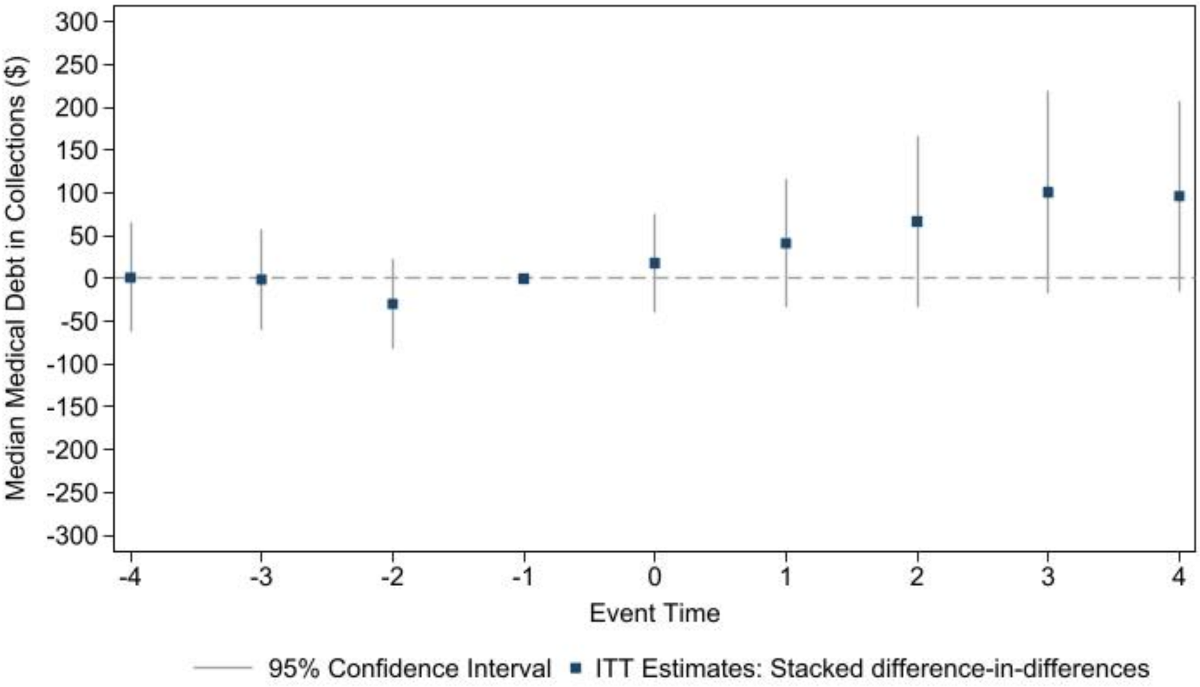

Notes: Figure displays event study regression results from the Cengiz et al. (2019) estimator comparing treated counties to never treated counties. We display event study coefficients 4 years prior to the first freestanding ED opening in a county and 4 years after the first freestanding ED opening, with 0 displaying the year of the opening.

2.2 Excluding independent Freestanding EDs

Although satellite freestanding emergency departments have existed for decades, similar facilities known as independent freestanding emergency departments entered the market in several states beginning with Texas in 2010.[5] Unlike their hospital affiliated counterparts, these facilities are not recognized by CMS as emergency departments and state regulation of these facilities varies. Because they are not CMS certified providers, independent freestanding EDs are unable to bill Medicare and Medicaid, thus choose to locate in areas with better payer mix and higher income marketing themselves as concierge medicine.[6] However, many of these facilities are out of network for major providers during our study period (before the enactment of the No Surprises Act) and due to the nature of their advertising practices could serve as a major source of confounding in our analysis. To explore whether our results are biased by the growth of independent freestanding EDs in the four states that have enabled their licensure we repeated our primary specification, excluding Colorado and Texas.[7] Delaware and Rhode Island were excluded from the study sample during restriction. Results from this analysis are displayed in eTable 2, below.

Overall, we find little difference when excluding independent freestanding EDs from our sample. The coefficients for median medical debt in collections and the share of the population with medical debt in collections are well within the 95% confidence interval of our primary results presented in Table 2 suggesting independent freestanding EDs are not a major source of confounding bias.

© 2025 Marthey D et al. *JAMA Network Open.*

eTable 2. Difference-in-Differences (DiD) Estimates of the Association Between Satellite Freestanding Emergency Departments Entry and County-Level Medical Debt, Robustness to Excluding States That Allow Independent Freestanding ED Licensure

	Median Medical Debt ($)		Share with Medical Debt		
	Coef.	SE	Coef.	SE (95% C.I.)	N
**DiD Estimate**	60.31	42.77 (-23.52-144.14)	0.010**	0.005 (<0.001-0.021)	13,695

Source: Centers for Medicare & Medicaid (CMS) Provider of Services (POS) Files and Urban Institute Credit Bureau Panel (2011-2021). **Notes**: *p<0.10, **p<0.05, ***p<0.01. Regressions are weighted by the average total population across the study period and robust standard errors are clustered on the county level. All models include year and county fixed effects and adjust for county-by-year social and demographic characteristics including the share of the total population by age, race/ethnicity, percent without health insurance coverage, percent unemployed, median household income, percent of the total population at or below 100% of the federal poverty level, and county urban/rural designation. Colorado and Texas are excluded from analysis as they are the only states which allow the licensure of independent freestanding EDs from our main study sample. See https://www.cms.gov/newsroom/press-releases/cms-issues-guidance-allowing-independent-freestanding-emergency-departments-provide-care-medicare.

© 2025 Marthey D et al. *JAMA Network Open.*

eReferences.

1. Center for Medicaid and State Operations, Survey and Certification Group. Requirements for Provider-based Off-campus Emergency Departments. Published online January 11, 2008. https://www.cms.gov/Medicare/Provider-Enrollment-and-Certification/SurveyCertificationGenInfo/downloads/scletter08-08.pdf

2. Goodman-Bacon A. Difference-in-differences with variation in treatment timing. *J Econom*. 2021;225(2):254-277. doi:10.1016/j.jeconom.2021.03.014

3. Callaway B, Sant'Anna PHC. Difference-in-Differences with multiple time periods. *J Econom*. 2021;225(2):200-230. doi:10.1016/j.jeconom.2020.12.001

4. Cengiz D, Dube A, Lindner A, Zipperer B. The Effect of Minimum Wages on Low-Wage Jobs*. *Q J Econ*. 2019;134(3):1405-1454. doi:10.1093/qje/qjz014

5. Marthey D, Ramy M, Ukert B. Who do freestanding emergency departments treat? Comparing Texas hospitals to satellite and independent freestanding departments in 2021 and 2022. *Health Serv Res*. Published online March 21, 2024:1475-6773.14304. doi:10.1111/1475-6773.14304

6. Dark C, Xu Y, Ho V. Freestanding Emergency Departments Preferentially Locate In Areas With Higher Household Income. *Health Aff Proj Hope*. 2017;36(10):1712-1719. doi:10.1377/hlthaff.2017.0235

7. Centers for Medicare and Medicaid Services. Guidance for Licensed Independent Freestanding Emergency Departments (EDs) to Participate in Medicare and Medicaid during the COVID-19 Public Health Emergency. Published online April 21, 2020. Accessed August 13, 2023. https://www.cms.gov/files/document/qso-20-27-hospital.pdf

© 2025 Marthey D et al. *JAMA Network Open.*
