# Supplementary material for: Medical Debt and Entry of Satellite Freestanding Emergency Departments
Source: JAMA Netw Open. 2025 Jul 23;8(7):e2522876. doi: 10.1001/jamanetworkopen.2025.22876 (PMC12287832; doi:10.1001/jamanetworkopen.2025.22876)
Supplement: Supplement 2. — Data Sharing Statement [file jamanetwopen-e2522876-s002.pdf]

Data Sharing Statement

Marthey. Medical Debt and Entry of Satellite Freestanding Emergency Departments. *JAMA Netw Open*. Published July 23, 2025. doi:10.1001/jamanetworkopen.2025.22876

Data
Data available: No

Additional Information
Explanation for why data not available: The data are publicly available.
